# Effect of Glycine on the Wet Carbonation of Steel Slag Used as a Cementitious Material

**DOI:** 10.3390/ma17020451

**Published:** 2024-01-17

**Authors:** Peiyu Cao, Xin Zhao, Yutong Wang, Zeyu Zhang, Jiaxiang Liu

**Affiliations:** Beijing Key Laboratory of Electrochemical Process and Technology for Materials, College of Materials Science and Engineering, Beijing University of Chemical Technology, Beijing 100029, China; thexmen@126.com (P.C.); zhaoxin1995hz@163.com (X.Z.); yutongwang16@163.com (Y.W.); 18513411068@163.com (Z.Z.)

**Keywords:** steel slag, wet carbonation, glycine, cementitious materials, cycling performance

## Abstract

The wet carbonation process of steel slag (SS) is envisaged to be an effective way to sequestrate CO_2_ and improve the properties of SS as a supplementary cementitious material. However, the carbonation process still struggles with having a low carbonation efficiency. This paper studied the effect of glycine on the accelerated carbonation of SS. The phase composition change of carbonated SS was analyzed via XRD, FT-IR, and TG–DTG. The carbonation process of SS is facilitated by the assistance of glycine, with which the carbonation degree is increased. After 60 min of carbonation, SS with glycine obtained a CO_2_ sequestration rate of 9.42%. Meanwhile, the carbonation reaction could decrease the content of free calcium oxide in SS. This significantly improves the soundness of SS–cement cementitious material, and the compressive strength of cementitious materials that contain carbonated SS with glycine is improved. Additionally, the cycling performance of glycine in the successive wet carbonation process of SS was investigated. Multicycle experiments via solvent recovery demonstrated that although the promotion effect of glycine was reduced after each cycle, compared with the SS–water system, the carbonation process could still be facilitated, demonstrating that successive wet carbonation via solvent recovery has considerable potential. Herein, we provide a new idea to facilitate the wet carbonation process of SS and improve the properties of SS–cement cementitious material.

## 1. Introduction

Steel slag (SS) is a by-product in the process of steel production, accounting for 15% to 20% of overall crude steel production [1]. In the 21st century, China’s SS production ranks first in the world, and its output exceeds 100 million tons per year, which is close to 50% of the world’s steel production [2]. Compared to wealthy countries, the application of SS in China is much lower. Most of China’s SS cannot be effectively utilized in a timely manner and storing it consumes a significant amount of land resources, causing serious pollution of the atmosphere and water resources. Toxic elements in SS, such as alkalis and heavy metals, can be released into the environment and affect living organisms [3]. Given China’s rising production of SS, the application of SS ought to be highly regarded. SS and cement have similar chemical compositions. SS contains mainly C_3_S (tricalcium silicate) and C_2_S (dicalcium silicate), which provide its hydraulic properties [4,5]. For this reason, using SS as a supplementary cementitious material (SCM) to partially replace cement is an effective method of utilizing SS. According to reports, 8% of global anthropogenic CO_2_ emissions are attributed to the manufacturing of Portland cement [6,7]. Thus, using SS as an SCM is the primary method for lowering CO_2_ emissions and the consumption of natural resources resulting from the manufacture of cement [8]. Applying SS as an SCM shows great potential, but its widespread application is constrained by its poor hydration activity and high volume expansion rate. The free calcium oxide (f-CaO) contained in SS will result in volume expansion during hydration, which will cause poor volume stability when using SS in civil engineering [9].

Carbonation treatments have attracted a lot of interest in recent years. The carbonation of SS can alleviate potential expansion by consuming large amounts of the free calcium oxide (f-CaO) in SS and converting it to thermodynamically stable carbonate [10,11]. Additionally, the calcium carbonate formed during the carbonation process has a beneficial effect on the mechanical strength of cementitious materials containing carbonated SS. Because of the high alkalinity of SS and the fact that the carbonate formed after the carbonation reaction remains thermodynamically stable, carbonation treatment of SS is regarded to be an effective way of permanent carbon sequestration as well as waste utilization [12,13]. In general, wet carbonation of SS reacts faster than dry carbonation [14]. However, wet carbonation of SS still faces the problem of low efficiency. The low efficiency of the wet carbonation process is due to slow carbonation kinetics. The carbonation reaction kinetics are constrained by slow Ca^2+^/Mg^2+^ leaching rates, low Ca^2+^/Mg^2+^ concentrations, and low carbonate/bicarbonate (CO_3_^2−^/HCO_3_^−^) concentrations in the solution due to limited CO_2_ solubility.

In order to enhance the carbonation behavior, various research efforts have been carried out to promote the leaching of calcium and magnesium ions. Additives like acids (e.g., HCl) and chelating agents (e.g., citrate), which bind Ca^2+^ or Mg^2+^ ions to promote leaching, have been extensively investigated [15,16,17]. However, some acids like humic acids bind Ca^2+^, forming insoluble humates and decreasing the amount of available Ca^2+^ ions [18]. Meanwhile, in order to facilitate the solubility of CO_2_, some CO_2_ capture solvents have been researched to accelerate CO_2_ absorption. For example, some amine-bearing solvents (e.g., monoethanolamine) are used to capture CO_2_ and can be regenerated via the formation of calcium carbonate [19,20]. Therefore, a major technological difficulty facing the wet carbonation of SS process is how to accomplish efficient leaching of calcium ions and CO_2_ capture in a simple process. So, there is a requirement for an ideal solvent that not only promotes the leaching of calcium ions but also facilitates the absorption of CO_2_ [21].

It is reported that glycine can act as a metal ion chelator, and the Ca-glycine stability constant is 2.19, which is close to calcium-chelating agent fumarate (2.00), adipate (2.19), and malonate (2.40). So, it can be used as an effective chelating agent to promote the leaching of calcium ions [22]. Meanwhile, glycine has proven to be a potential solvent for promoting CO_2_ absorption. Also, glycine is regenerated during calcium carbonate production; therefore, glycine has the potential to be reused through solvent recovery [23]. For wet carbonation methods, solvent recovery and reuse is also crucial. Reusing solvents has the potential to increase financial benefits and reducing energy consumption [24]. However, there is still no research that investigates glycine-assisted wet carbonation of SS. Meanwhile, the effect of carbonated SS with glycine on the properties of SS–cement cementitious materials should be explored. Considering these factors, this paper attempts to use glycine to promote the carbonation process of SS while improving the soundness and mechanical strength of SS–cement cementitious materials. Also, the cycling performance of glycine in the successive wet carbonation process of SS was investigated.

In this work, we attempted to use glycine as an additive for SS wet carbonation and systematically investigated the evolution of the carbonated phase using XRD, FT-IR, and TGA methods. Then, we partly replaced cement with carbonated SS for the preparation of SS–cement cementitious materials, and research was conducted on how the carbonated SS affected the compressive strength and soundness of the SS–cement cementitious materials. Finally, three cycle experiments were performed to examine the solvent recovery performance of successive wet carbonation of SS when glycine was used as a wet carbonation additive.

## 2. Materials and Methods

### 2.1. Materials

**Figure 1:**
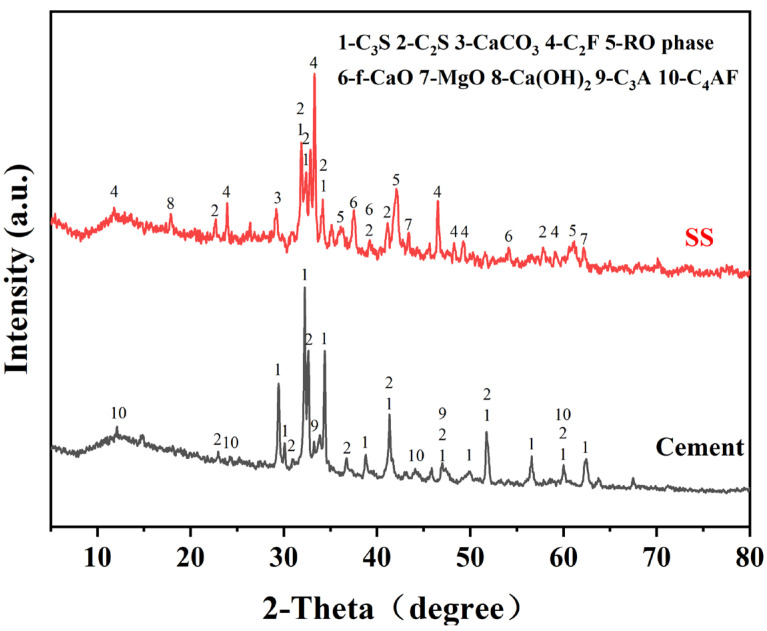
XRD patterns of the SS and cement. (C_3_S: tricalcium silicate, C_2_S: dicalcium silicate, C_2_F: dicalcium ferrite, f-CaO: free calcium oxide, C_3_A: tricalcium aluminate, C_4_AF: tetracalcium aluminoferrite).

**Figure 2:**
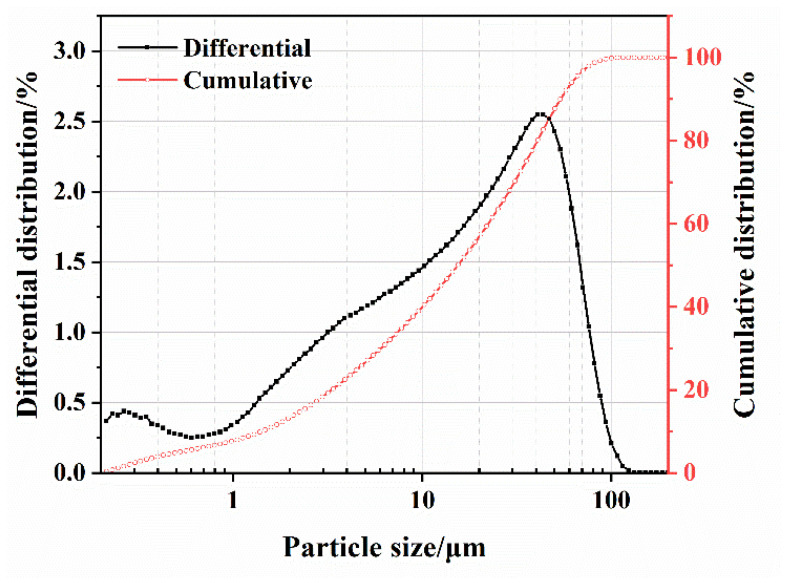
Particle size distribution of the SS powder.

The SS was provided by Shougang Jingtang Iron and Steel United Co., Ltd. (Tangshan, China). Figure 1 displays the mineral compositions of the cement and SS, and Figure 2 depicts the particle size distribution of the SS and cement powder.

Glycine of 99% purity was purchased from Shanghai Macklin Biochemical Co., Ltd. (Shanghai, China). CO_2_ gas with a purity of 99.9% was supplied by Beijing Hongmao Shuntong Energy Technology Co., Ltd. (Beijing, China).

### 2.2. Methods

#### 2.2.1. Wet Carbonation of SS

**Figure 3:**
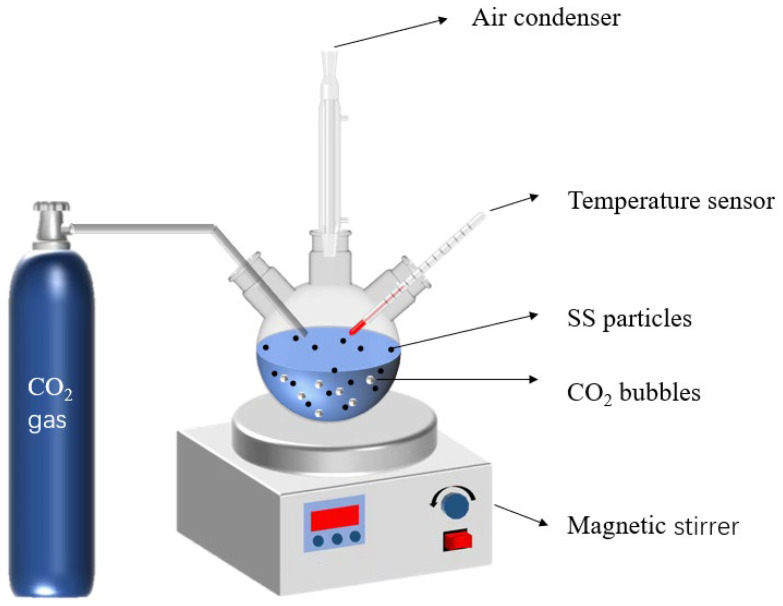
Model of the carbonation reaction experimental device.

In order to examine the effect of glycine concentration on the carbonation degree of SS, glycine concentrations were determined at 0.05 mol/L, 0.1 mol/L, and 0.25 mol/L, with a solid–liquid ratio of 1:5, and carbonation temperature at 40 °C. Shown in Figure 3 is the device for the wet carbonation reaction. To promote the leaching of calcium ions, the SS was first added to 200 mL of glycine solution with a solid/liquid ratio of 1:5 and stirred at 40 °C for 30 min for pre-leaching. CO_2_ gas of 99% purity was introduced to the solution for 60 min with a flow rate of 200 mL/min. The solid deposits were dried following centrifugation, and underwent XRD, FT-IR, and TG–DTG tests. An identical carbonation experiment was conducted using DI water (deionized water) without glycine for the control group. The four groups of carbonated samples were named G0CSS-60, G1CSS-60, G2CSS-60, and G3CSS-60. In our experiments, we made sure that all of the raw material used came from the same batch of steel slag and conducted the experiments in the same month.

To study how glycine affects the carbonation process of SS, the SS was first added to 200 mL of the 0.25 mol/L glycine solution at a solid–liquid ratio of 1:5 and stirred at 40 °C for 30 min for pre-leaching. The 99% CO_2_ gas was injected into the solution with a flow rate of 200 mL/min. The carbonation time was set to 15 min, 30 min, and 60 min, respectively. The solid deposits were dried following centrifugation, and we then performed XRD, FT-IR, and TG–DTG analysis. The three groups of carbonated samples were named G3CSS-15, G3CSS-30 and G3CSS-60.

Table 1 shows the proportions used in each wet carbonation experiment conducted under various experimental conditions.

#### 2.2.2. Preparation of Carbonated SS–Cement Cementitious Material

SS was carbonated via wet carbonation with different concentrations of glycine. Mortar samples were then prepared by blending the carbonated SS with cement (with SS accounting for 10% of the cementitious material) and standard sand at a water–binder ratio of 0.5. According to GB/T 17671–2021 [25], mortar samples of a 40 mm × 40 mm × 160 mm size were made. The mortar samples were then moved into a constant temperature and humidity curing box (temperature 20 ± 1 °C, relative humidity 95%). After 24 h of curing, the mortar samples were demolded and placed in a thermostatic water curing box (20 °C). After 3, 7, and 28 days of water curing, respectively, the mortar samples were tested for compressive strength. In our experiments, we made sure that the same instruments were used for testing and parallel experiments were conducted.

In order to evaluate the soundness of the carbonated SS, paste samples were prepared according to the ratio of mortar samples. According to GB/T 1346–2011 [26], the paste sample was loaded into Le Chatelier needles and cured for 24 h; the expansion value of the needles was tested using a Le Chatelier soundness test.

In order to learn more about how glycine affects the strength development of the samples, the amount of chemically bound water in each specimen was calculated. The experimental samples were taken out after 28 days of hydration, and the hydration was ceased with water-free ethanol. After 24 h, the samples were dried at 105 °C for 24 h, and then ground into powders for TG–DTG testing.

The chemically bound water content was calculated using the following formula:

(1) $$\mathrm{CW}\;(\mathrm{wt}.\%)=\;\frac{M105\;{}^{\circ}C-M300\;{}^{\circ}C}{M\;sample}\times 100$$

where M is the mass of the sample at a given temperature.

### 2.3. Multicycle Carbonation Experiment

**Figure 4:**
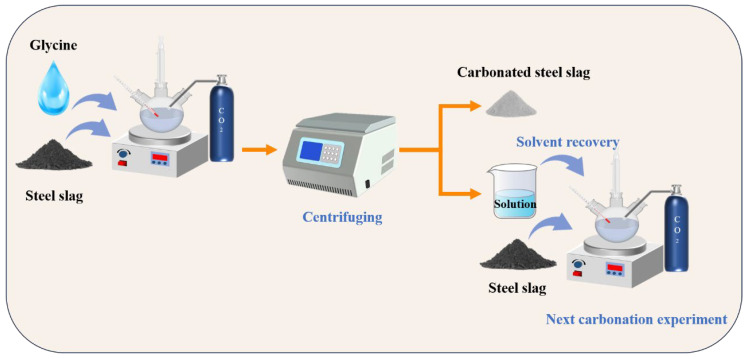
Schematic illustration of successive wet carbonation experiments.

To further study the cycling performance of glycine in the successive wet carbonation process of SS, three cycles of carbonation experiments were performed. Figure 4 illustrates the successive wet carbonation procedure. Firstly, the SS was added to 200 mL of the 0.25 mol/L glycine solution at a solid/liquid ratio of 1:5 for 30 min for pre-leaching. Then, the suspension was continuously injected with 99% CO_2_ for 60 min with flow rate of 200 mL/min. At the end of the carbonation experiment, the solids and solution were separated with centrifugation, then the solution was recycled and supplemented with DI water (deionized water) until the overall volume of the solution reached 200 mL, which was used as a solution for the next carbonation process. The same amount of SS was then added into solution and then the experiment repeated. The remaining solids from each carbonation experiment were utilized for XRD and TG–DTG testing after drying. The three groups of these samples were named Cycle1, Cycle2, and Cycle3.

## 3. Results and Discussion

### 3.1. Reaction and Mechanism during Carbonation of SS with Glycine

To be specific, slow Ca^2+^/Mg^2+^ leaching, a low Ca^2+^/Mg^2+^ concentration, limited CO_2_ solubility, and low carbonate/bicarbonate (CO_3_^2−^/HCO_3_^−^) concentrations in the water all hampered the reaction kinetics of CO_2_ mineralization. When SS is dissolved in water, the calcium-bearing mineral phase continues to leach Ca^2+^, and when CO_2_ is injected into the solution, it dissolves and converts into carbonate and forms CaCO_3_ precipitate. Specifically, the constant leaching of Ca^2+^ from the calcium-bearing mineral phase and the solubility of CO_2_ are essential to this process. Meanwhile, the CaCO_3_ consistently generated during the carbonation reaction may be deposited on the SS surface, perhaps impeding the leaching of internal calcium and reducing the reaction rate [27]. Glycine can chelate Ca^2+^ and promote Ca^2+^ extraction and leaching, thus increasing the rate and extent of carbonation.

The proposed chemical reaction pathways for the leaching–carbonation process are given in Table 2. In the leaching step, The calcium-bearing mineral phase will continue to leach Ca^2+^ by reacting with water or the glycine solution (reactions 1–2). Glycine promotes Ca^2+^ leaching by dissociating protons and chelating Ca^2+^ and produces gly^—^ (deprotonated glycine). In the carbonation step, after CO_2_ is introduced into the leachate, the CO_2_ dissolves and generates carbonate and bicarbonate. Additionally, the presence of gly^—^ (deprotonated glycine) can provide an additional reaction pathway for CO_2_ absorption by reacting with CO_2_ through reaction 7. Since the kinetics of this process is faster than the CO_2_ hydration pathway, the presence of glycine increases the concentration of carbonate and bicarbonate in the aqueous phase, thus facilitating the carbonation reaction [28,29,30]. Calcium carbonate will continue to be produced through reactions 6 and 8, and glycine can be regenerated and reused through solvent recovery [31].

### 3.2. XRD Analysis of Carbonated SS

**Figure 5:**
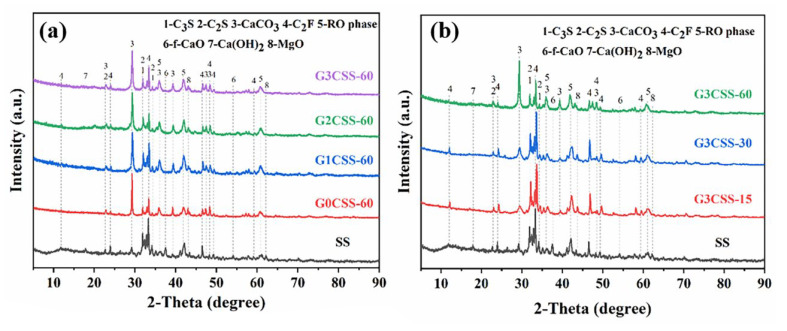
XRD pattern of SS and carbonated SS with different carbonation conditions (G3CSS-15: carbonation time is 15 min; G3CSS-60: carbonation time is 60 min). (**a**) XRD patterns of carbonated SS added with different concentrations of glycine; (**b**) XRD patterns of carbonated SS with different carbonation time.

Figure 5 depicts the XRD pattern of the SS and the carbonated SS with glycine. As can be seen from Figure 5a, the main mineral phases in SS include di-calcium silicate (C_2_S), tri-calcium silicate (C_3_S), free calcium oxide (f-CaO), portlandite (Ca(OH)_2_), RO phase, di-calcium ferrite (C_2_F), etc. The main products of the carbonation of SS are calcium carbonate and calcite, regardless of the concentration of glycine. This may be attributed to the fact that this experiment was designed to be reacted in a solution, while calcite is more easily formed in wet carbonation and aragonite is more easily formed in dry carbonation [32]. As shown in Figure 5b, the diffraction peaks of the f-CaO phase disappeared with the carbonation reaction, indicating that carbonation could efficiently reduce the f-CaO phase within the SS and convert it into stable CaCO_3_ to improve the volume stability. It was also observed that during the wet carbonation reaction, C_2_S and C_3_S also react with CO_2_, and its diffraction peak intensity decreased after carbonation [33]. However, phases with a high iron content were still present in the samples after 60 min of carbonation, indicating that they exhibit low reactivity with CO_2_.

**Figure 6:**
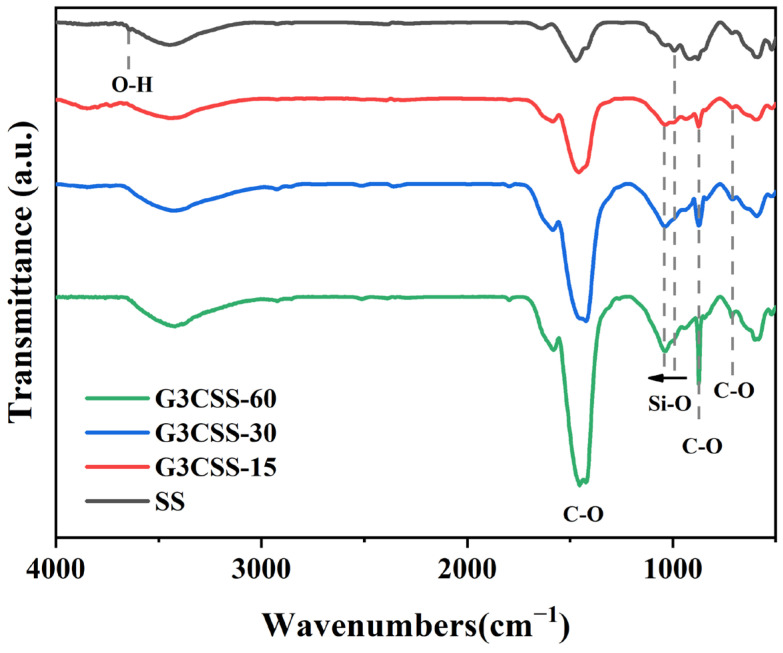
The FTIR spectra of GCSS across different carbonation times.

In order to further characterize the chemical bonds of the carbonation products, FTIR analyses were performed on the carbonated SS, and the resulting spectra are displayed in Figure 6. The peaks at 3641 cm^−1^ correspond to the H–O stretching vibrations of portlandite, which disappeared after carbonation [34]. The new peaks at 712 cm^−1^, 872 cm^−1^, and 1419 cm^−1^ are the calcite in carbonate. No traces of vaterite and aragonite can be observed, consistent with the XRD analyses. The broad absorption peak in the 900–1200 cm^−1^ range corresponds to the Si–O bond vibration in SS. Particularly, the peak at around 998 cm^−1^ is caused by the asymmetric stretching of Si–O bonds present in C-S-H gel [35]. During the carbonation process, the peak of Si–O bonds between 900 cm^−1^ and 1200 cm^−1^ continued to move up to higher wavenumbers, indicating that portlandite and C-S-H gel could release calcium ions and form CaCO_3_ during wet carbonation, resulting in an increased polymerization of silicate and production of amorphous silica gel [36].

### 3.3. TG–DTG Analysis of Carbonated SS

**Figure 7:**
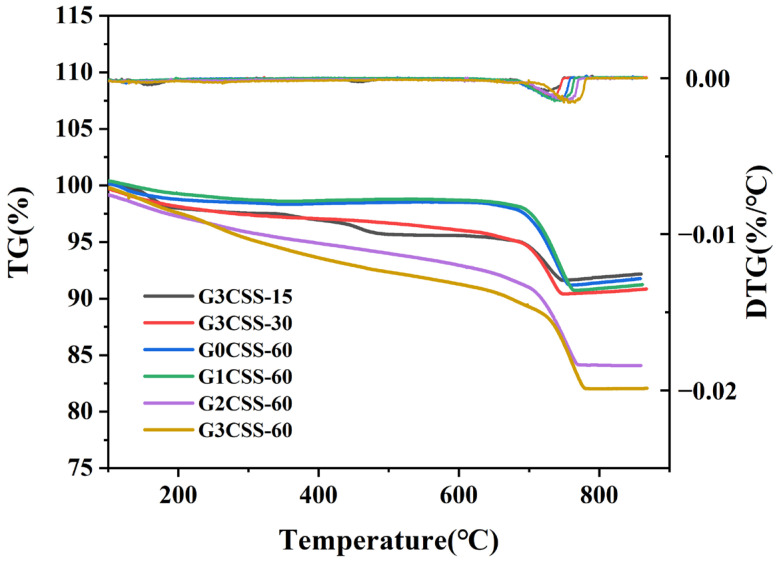
TG and DTG curves of GCSS under various carbonation conditions.

Figure 7 displays the TG–DTG curves for each sample. It can be determined from Figure 7 that the main weight loss interval appears between 600 and 800 °C, which corresponds to the decomposition of CaCO_3_ [32]. It can be seen that the carbonation process is facilitated in the SS–glycine system, and as the glycine addition increases, the degree of carbonation of SS is promoted. The CO_2_ sequestered and CaCO_3_ formed for different carbonated SS samples are calculated and presented in Table 3. As can be seen, wet carbonation of the G3CSS-60 sample in 60 min can absorb 2.231 g more CO_2_ per 100 g sample and produce 5.07 g more CaCO_3_ per 100 g sample than the G0CSS-60 (control group) sample.

### 3.4. Utilization of Carbonated SS in SS–Cement Cementitious Materials

#### 3.4.1. Compressive Strength of SS–Cement Cementitious Materials

**Figure 8:**
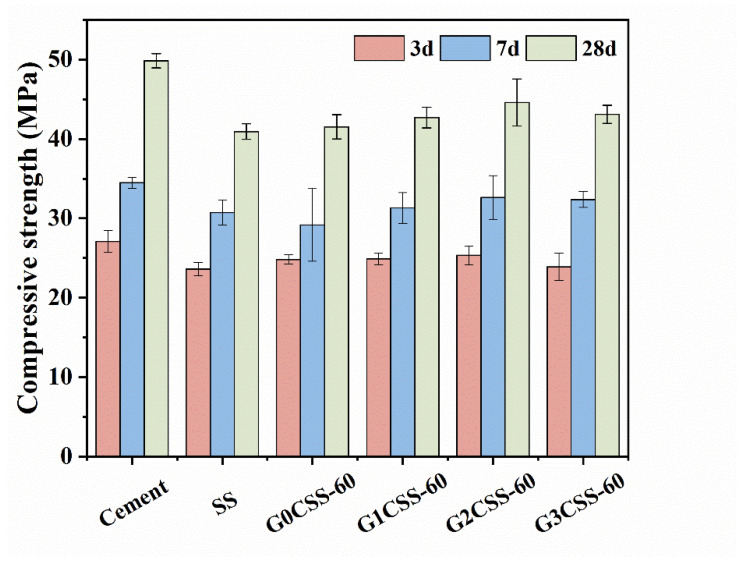
Compressive strength of different mortar samples.

Figure 8 presents the compressive strength of different mortar samples at 3, 7, and 28 days. Partially replacing cement with SS leads to a significant reduction in the compressive strength due to the poor hydration reactivity of SS compared to pure cement mortar samples [37]. The main mineral compositions in SS were β-C_2_S and γ-C_2_S, of which γ-C_2_S has a poor hydration reactivity. Additionally, the C_2_F, Fe-containing solid solutions, and RO phase of the SS all showed limited hydration reactivity, which may slightly contribute to the strength development of the cement mortar [38].

It is worth noting that at different ages, compared to the mortar sample containing uncarbonated SS, the mortar samples that contain carbonated SS had higher compressive strengths. Besides, mortar samples that contained carbonated SS with glycine experienced further increases in their compressive strength. Nonetheless, the 28-day compressive strength growth rate did not increase with the addition of glycine but reached its maximum value at a glycine addition of 0.1 mol/L (the G2CSS-60 group), with which the maximum improvement was 8.48%. This can be attributed to the fact that the addition of carbonated SS with glycine contains more fine CaCO_3_, which acts as a nucleation site for the hydration products, lowering the nucleation barrier and accelerating the hydration of the cement [39]. In the later stage, calcium carbonate can also react with the aluminum phase in the cement to form Mc with a certain cementitious ability, so that the compressive strength of cementitious materials can be enhanced. This will be thoroughly discussed later.

**Figure 9:**
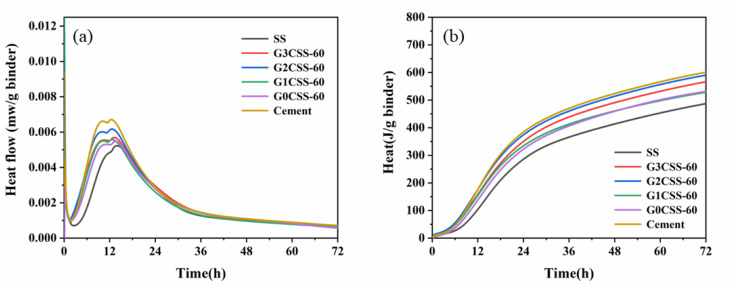
Effect of carbonation treatments on (**a**) heat flow and (**b**) the cumulative heat of blended paste samples.

Figure 9 shows the heat flow and cumulative heat of the SS cement paste samples. From Figure 9a, it can be noticed that the exothermic peak each the paste sample decreases after being added to SS. As the hydration activity of SS is poor, it acts as an inert material in the early stages. At the same time, the exothermic peak shifts to the right, which proves that the SS impedes the cement’s hydration [40]. However, the early hydration of the paste samples that contain carbonated SS was accelerated, indicating that adding carbonated SS introduces more calcite as a nucleation site. The increase in hydrate nucleation sites provides an additional nucleation surface, leading to faster growth of hydration products [41]. Figure 9b shows the cumulative heat of each paste sample and as can be seen in this figure, using SS or carbonated SS as a cement substitute reduces the overall cumulative heat of the hydration process because of the lower cement content and clinker dilution effect [40]. Except for the control group of cement, the paste samples containing G2CSS-60 exhibited the highest cumulative heat, as reflected by the compressive strength results.

**Figure 10:**
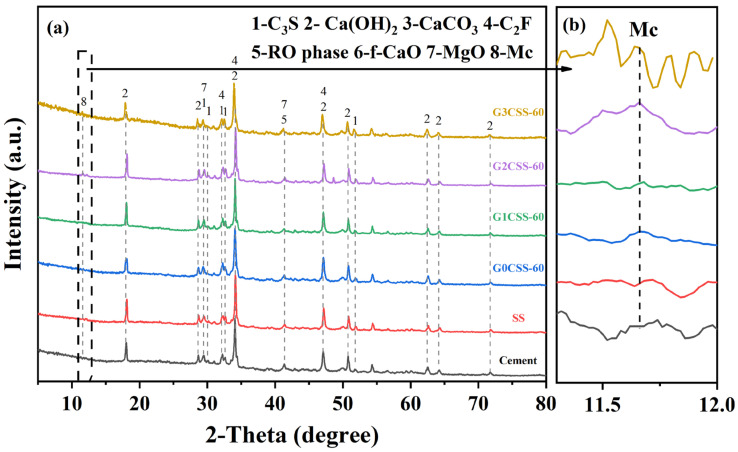
(**a**) XRD patterns of the paste samples at 28 days. (**b**) Magnified pattern in the 2θ range of 11.3°–12°, showing the diffraction peak of Mc.

The composition of the 28-day paste samples was investigated using TG–DTG and XRD tests. Figure 10 shows the XRD test results of each paste sample at the age of 28 days, and it is found that the main phases of each sample after 28 days of hydration were the same, which consisted primarily of unhydrated calcium silicate, Ca(OH)_2_, calcium carbonate (CaCO_3_), C_2_F, RO phase, and MgO. It is noteworthy that the diffraction peaks of monocarboaluminate (Mc, 2θ = 11.7) appear in the G2CSS-60 and G3CSS-60 samples. This might be attributed to the higher content of calcium carbonate in these two samples and further reactions with the aluminum phase to form Mc.

**Figure 11:**
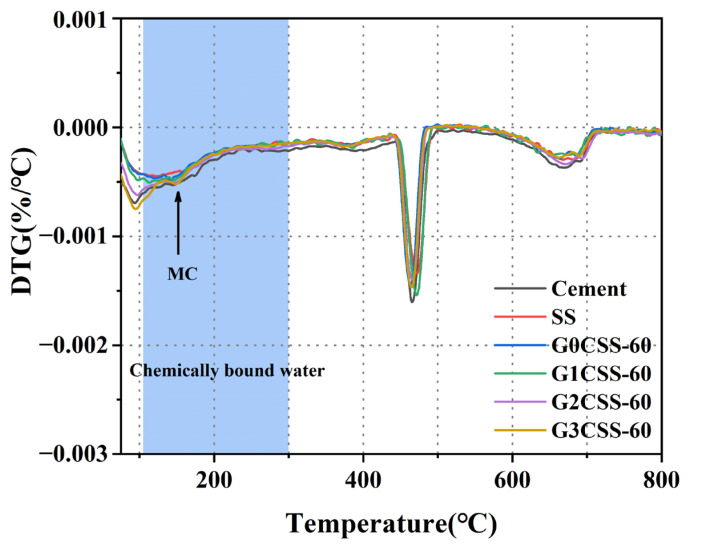
DTG curves of paste samples at 28 days.

The DTG curves of each paste sample at 28 days are shown in Figure 11. It was found that a new endothermic peak at 150 °C appeared in the G2CSS-60 and G3CSS-60 samples, which was attributed to the loss of water in the Mc, confirming the formation of the Mc phase, which is consistent with the results of the XRD test [42,43]. According to previous studies, monocarboaluminate hydrates are beneficial to the compressive strength development of cementitious materials [44,45].

**Figure 12:**
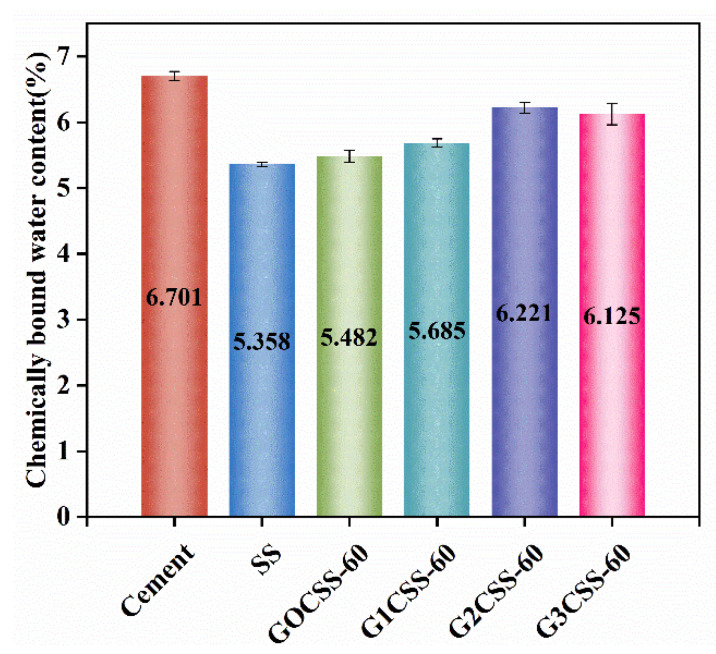
Chemically bound water content of different paste samples at 28 days.

In order to learn more about how glycine affects sample strength development, the chemically bound water content of each sample was tested. The content of chemically bound water at a certain age can reflect the degree of hydration reactions in cement-based materials [46]. In the temperature range of 105−300 °C, hydration products like AFT and CSH decompose, so the amount of chemically bound water in paste samples can be calculated based on the mass loss from 105 to 300 °C [32]. These test results are presented in Figure 12, among which the G2CSS-60 group was the highest, indicating that it had the highest hydration degree. Accordingly, the G2CSS-60 group obtained the highest 28-day compressive strength.

**Figure 13:**
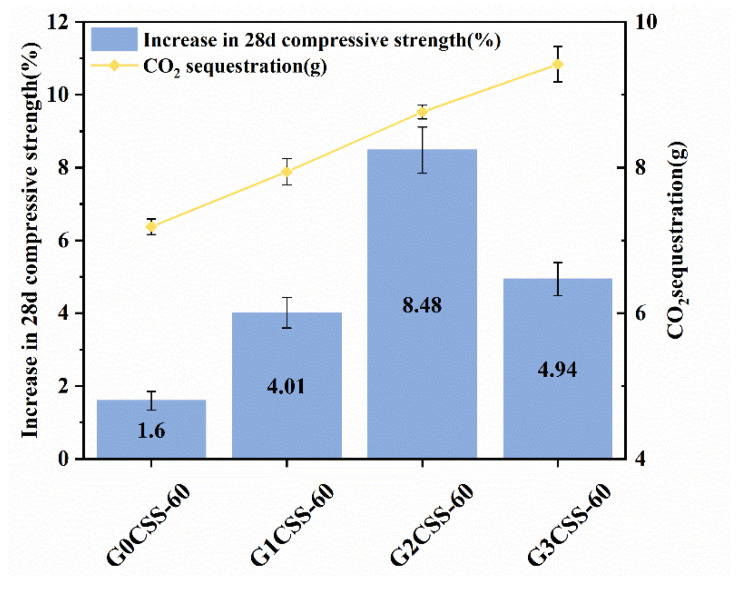
Compressive strength increase rates of mortar samples supplemented with carbonated SS of different carbonation conditions (or with uncarbonated SS as a control group) over 28 days.

Depicted in Figure 13 is the 28-day compressive strength increase rates of mortar samples containing carbonated SS with different carbonation conditions, and the blank group is the mortar sample containing uncarbonated SS. With increasing CO_2_ sequestration, the 28-day compressive strength increase rate increased and then decreased, reaching its maximum with the glycine concentration of 0.1 ml/L (G2CSS-60 group). By affecting the carbonation properties of SS, glycine affects the hydration process of cementitious materials, which ultimately results in strength growth. The carbonation degree of SS improves with increases in the glycine concentration, and more calcium carbonate is produced. In the early stages of hydration, the calcium carbonate acts as a nucleation site for the hydration products, which lowers the nucleation barrier and accelerates the hydration of the cement. In the later stages, calcium carbonate can also react with the aluminum phase in the cement to form Mc with certain cementitious abilities, which positively affects the compressive strength development of the cementitious material.

However, as the concentration of glycine continues to increase, the carbonation degree of SS further increases, and the cementitious phases in SS such as C_3_S and C_2_S overreact with CO_2_, resulting in a larger consumption of cementitious phases. Thus, the negative effect exceeds the positive effect and ultimately, the 28-day compressive strength growth rate decrease [33]. Therefore, the concentration of glycine and other carbonation parameters should be selected appropriately for better application in cementitious materials.

#### 3.4.2. Soundness of SS–Cement Cementitious Materials

The f-CaO contained in SS will hydrate and cause large volume expansion during the hydration process, which will cause poor volume stability when using SS in civil engineering [47]. However, through the carbonation process, these substances in the SS can be converted to thermodynamically stable carbonate products, thereby effectively enhancing the soundness of the SS. In this study, expansion values of the paste samples that contain carbonated SS with glycine were measured with the Le Chatelier soundness test. These results are presented in Table 4. The results of the test demonstrate that the expansion values of the paste samples decrease after carbonation treatment. As the concentration of glycine increases, the carbonation degree increases, and the expansion values of the paste samples further decrease to 0 mm. The carbonation process consumes a significant percentage of the f-CaO within the SS, which improves the volume stability of the paste samples.

### 3.5. Multiple Cycles of Wet Carbonation of SS with Glycine

**Figure 14:**
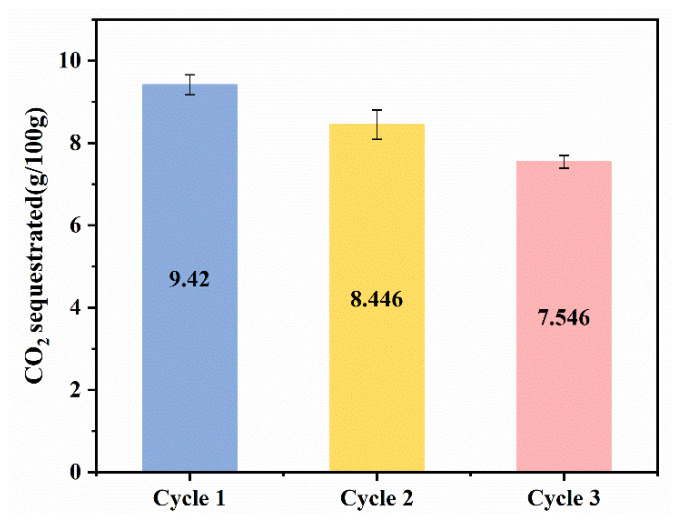
CO_2_ sequestration of multicycle carbonation experiments.

Most of the previous studies only changed the experimental conditions to conduct single carbonation experiments and generally focused on the properties of the solid products produced after solid–liquid separation at the end of the carbonation experiments, while little attention has been paid to the treatment of the remaining solution. Meanwhile, as mentioned in Section 3.1, glycine is regenerated during the carbonation process and can be used again via solvent recovery at the end of carbonation experiments. So, three cyclic experiments were performed to study the cycling performance of glycine in our successive wet carbonation experiments of SS. As demonstrated by Figure 14, as the cyclic experiment progressed, the amount of CO_2_ sequestration gradually decreased in each cycle. This may be due to the loss of glycine in the carbonation process, which reduces the carbonation’s promoting effect. Although the promotion effect of carbonated SS with glycine is gradually weakened, when compared with the SS–water system, the carbonation process is still enhanced.

**Figure 15:**
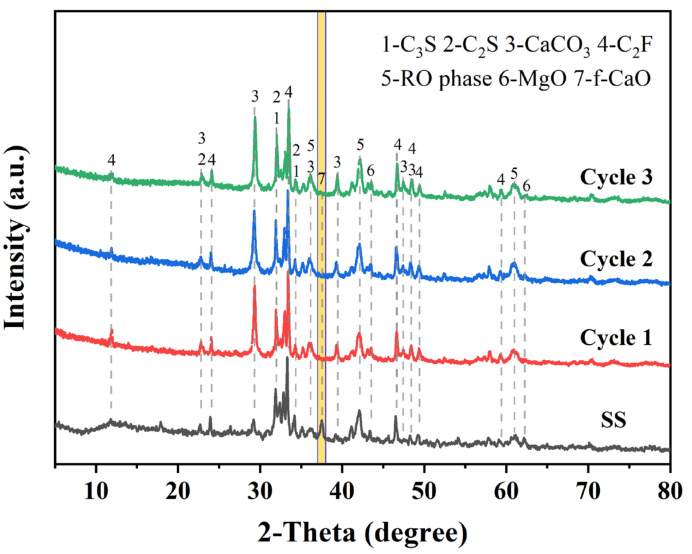
XRD patterns of carbonated SS obtained from multicycle carbonation experiments. (The yellow bar marks the diffraction peak of f-CaO).

In addition, the carbonated SS samples produced through our three cyclic experiments have stable properties. As shown in Figure 15, the XRD test results confirm that the carbonated SS obtained from the three cyclic experiments contains calcite as a carbonation product and that the f-CaO in SS was effectively eliminated. This indicates that glycine-assisted successive wet carbonation of SS possess considerable potential.

## 4. Conclusions

This research studied the process and mechanism of glycine-assisted SS wet carbonation and partially replacing cement with carbonated SS for the preparation of cementitious materials. Additionally, the feasibility of solvent recovery in the successive wet carbonation process of SS was discussed. The primary findings are as follows:

1. The addition of glycine facilitates the carbonation process of SS and the carbonation efficiency is increased. The carbonation degree of SS improves with increasing glycine additions. When the concentration of glycine is 0.25 mol/L and the carbonation time is 1 h, the CO_2_ sequestration rate of SS is 9.42%, while for the sample without glycine, it was 7.189%. The carbonation products are mainly calcium carbonate with crystal-type calcite.
2. After carbonation treatment, the expansive-phase f-CaO is reacted and convert into thermodynamically stable carbonate products, thus the soundness of SS is efficiently improved. The 28-day compressive strength of the cementitious material that contained carbonated SS with glycine was improved compared to the sample that contained uncarbonated SS. The maximum increase was 8.48%.
3. Glycine promotes the carbonation process of SS and can be reused during successive wet carbonation of SS. Multicycle experiments via solvent recovery demonstrated that although the promotional effect of glycine was reduced after each cycle, compared with the SS–water system, the carbonation process was still facilitated. The carbonated SS obtained from each experiment contains calcite as a carbonation product and the f-CaO in the SS was effectively eliminated.

## Figures and Tables

**Table 1:** The proportion used in the SS wet carbonation experiment with glycine.

Sample	Concentration of Glycine (mol/L)	Carbonation Time (h)	Solid–Liquid Ratio
G0CSS-60	—	1	1:5
G1CSS-60	0.05	1	1:5
G2CSS-60	0.1	1	1:5
G3CSS-60	0.25	1	1:5
G3CSS-30	0.25	0.5	1:5
G3CSS-15	0.25	0.25	1:5

**Table 2:** Proposed reaction pathways in the leaching–carbonation process.

Steps	Reaction NO.	Reactions
Leaching	1	Calcium-bearing mineral phase ↔ Ca^2+^ + OH^−^
	2	Calcium-bearing mineral phase + 2NH_2_CH_2_COOH ↔ 2NH_2_CH_2_COO^−^ + Ca^2+^ + H_2_O
Carbonation	3	CO_2_ (aq) + H_2_O ↔ H_2_CO_3_
	4	H_2_CO_3_ + OH^−^ ↔ HCO_3_^−^
	5	HCO_3_^−^ + OH^−^ ↔ CO_3_^2−^
	6	Ca^2+^ + CO_3_^2−^ ↔ CaCO_3_↓
	7	2NH_2_CH_2_COO^−^ + CO_2_ ↔ NHCOO^−^CH_2_COO^−^ + NH_3_^+^ CH_2_COO^−^
	8	NHCOO^−^CH_2_COO^−^ + NH_3_^+^ CH_2_COO^−^ + Ca^2+^ + H_2_O ↔ CaCO_3_↓ + 2NH_2_CH_2_COOH

**Table 3:** Amount of CO_2_ sequestered and CaCO_3_ formed after wet carbonation (g/100 g sample).

Sample	CO_2_ Sequestration	Estimated Mass of CaCO_3_ Formed
G3CSS-15	3.687	8.38
G3CSS-30	5.49	12.477
G0CSS-60	7.189	16.339
G1CSS-60	7.943	18.059
G2CSS-60	8.765	19.92
G3CSS-60	9.42	21.409

**Table 4:** Expansion values of paste samples.

Sample	Expansion Value (mm)
SS	0.5
G0CSS-60	0.17
G1CSS-60	0.17
G2CSS-60	0
G3CSS-60	0

## Data Availability

The data presented in this study are available on request from the corresponding author.
